# Development of Fish Immunity and the Role of β-Glucan in Immune Responses

**DOI:** 10.3390/molecules25225378

**Published:** 2020-11-17

**Authors:** Marianna V. Rodrigues, Fábio S. Zanuzzo, João Fernando A. Koch, Carlos Alberto F. de Oliveira, Petr Sima, Vaclav Vetvicka

**Affiliations:** 1 Biotechnology Institute, UNESP-São Paulo State University (Unesp), Alameda das Tecomarias s/n, 18607-440 Botucatu, São Paulo, Brazil; mvazrodrigues@gmail.com; 2 Department of Ocean Sciences, Memorial University, St. John’s, NL A1C5S7, Canada; fabioz@mun.ca; 3 Department of Research and Development, Biorigin Company, Fazenda São José s/n, 17290-000 Macatuba, São Paulo, Brazil; joao.koch@biorigin.net (J.F.A.K.); carlos.food.eng@gmail.com (C.A.F.d.O.); 4 Institute of Microbiology, Czech Academy of Sciences, 14220 Prague, Czech Republic; sima@biomed.cas.cz; 5 Department of Pathology, University of Louisville, Louisville, KY 40292, USA

**Keywords:** fish, glucan, immunity, feeding, infection, health

## Abstract

Administration of β-glucans through various routes, including immersion, dietary inclusion, or injection, have been found to stimulate various facets of immune responses, such as resistance to infections and resistance to environmental stress. β-Glucans used as an immunomodulatory food supplement have been found beneficial in eliciting immunity in commercial aquaculture. Despite extensive research involving more than 3000 published studies, knowledge of the receptors involved in recognition of β-glucans, their downstream signaling, and overall mechanisms of action is still lacking. The aim of this review is to summarize and discuss what is currently known about of the use of β-glucans in fish.

## 1. Introduction

Aquaculture is quickly becoming a crucial food-producing sector. One of the consistent problems is health management, as ever-increasing fish density results in elevated stress often leading to outbreaks of deadly diseases. Besides antibiotics, which use is increasingly monitored and often prohibited, the need to establish new ways of potentiation of immune reaction is clear. One option is the use of β-glucan, which has been tested in fish for decades.

The first report approaching the β-glucan molecule was published in 1946 by Dimler, et al. [1], and the authors successfully isolated the molecule d-glucosan β (1,4) (1,6) from starch. However, the first robust scientific evidence showing that β-glucan affects the immunity was published almost 20 years later in the journal, *Science*. Wooles and Diluzio [2] injected mice with β-glucan and observed a higher hyperphagocytic activity of the reticuloendothelial system, and the authors associated this response to an increase in the primary and secondary immune responses of mice to sheep erythrocytes. Ever since, the effects of β-glucan have been extensively studied, and a well-known immunomodulator suitable for bath treatment, injection, and dietary administration in vertebrates. For example, the positive effects of β-glucans in immunity have been shown in humans [3], dogs [4], pigs [4,5], cattle [6], horses [7], sheep [8], chickens [9], frogs [10], fish [11]; invertebrates, such as shrimp [12] and crab [13]; and insects, such as bees [14] and drosophila [15].

The effect of β-glucans in the immunity of a wide number of species in general is related to the conserved pathways of defense reactions among the vertebrate species, more specifically related to recognition of pathogen-associated molecular patterns [16]. In this context, fish as the first vertebrate group appearing in evolution after adaptive radiation during the Devonian Period, play an important role in the evolution of the immune responses, and an apparent crossroads between the innate immune response and the appearance of the adaptive immune response [17].

Glucan plays a particularly important role in aquaculture as demand for fish and shrimp increases. Aquaculture is plagued with several problems from environmental pollution to fish diseases, and it is imperative to improve health of farm animals. This is true not only from the commercial point of view, but also from the point of minimalizing the spread of diseases to the outside world. Farming of species can be a vector for disease proliferation in the wild environment. Disease transfer in salmon aquaculture is perhaps the most reported instance of this phenomenon. The disease, infectious salmon anemia, first appeared in Chile in the 1990s, and has since been noted in other environments around the world. The search for natural substances used as dietary feed supplements is ongoing.

In this review, we approached different aspects of the development of the fish immunity system and how it can be associated with the effects of β-glucan. In addition, we discussed the application and mechanism of action of β-glucans in fish.

## 2. The Use of Immunomodulators Compounds

The industry of animal protein production is growing exponentially, and it is inevitable that intensive animal production stresses the animals by confinement, transport, and handling, creating a physiological condition characterized by suppressed immunity and consequently higher susceptibility to disease [18]. Particularly, in the aquaculture sector, the farmer cannot always visually assess the fish and very often the perception of disease outbreaks is a challenge. The indiscriminate use of antibiotic to prevent these outbreaks has resulted in the emergence of several resistant pathogens in aquaculture [19], impeding the development and sustainability of the industry worldwide [20]. In this context, many immunostimulant compounds, such as bacterial lipopolysaccharides (LPS), mannooligosaccharides, vitamins, minerals, and animal and plant extracts, have been widely investigated to enhance fish immunity and protect against disease [18,21,22]. Among these compounds, β-glucans stand out and their role as a biologically active immunomodulator in fish has been well documented.

## 3. The Molecule of β-Glucans and Their Effects

“Glucan” is the common name given to a group of polysaccharide polymers, classified based on interchain linkages as either α or β linked. β-Glucans obtained from different sources often have different primary structures and conformations. The primary structure is defined by the glycosidic bond type, as well as degrees of branching and polymerization, while the conformation of β-glucans often presents as a random coil, single helix, or triple helix, and is affected by the primary structure, intermolecular force, temperature, and solvent [23].

β-Glucans are widely distributed in bacteria, algae, fungi, and plants, with different structural types (see Barsanti et al. [24]). Their structure is comprised of a main chain of β (1,3) and/or β (1,4)-d-glucopyranosyl units in nonrepeating but nonrandom order, with side chains of varying lengths [25]. In this context, the different β-glucan molecules may differ in their activity/effectiveness as immunomodulator, and even molecules with similar structures, molecular weights, and solution conformations can differ markedly. Many studies have reported correlations between β-glucan effectiveness and molecular structure, size, branching frequency, structural modification, conformation, and their solubility; however, it is risky to make generalizations due the often-contradictory data [26]. In addition, numerous concentrations and routes of administration have been tested including oral applications. Despite extensive investigations, consensus on the source, size, and other biochemical or physicochemical properties of β-glucans has not been achieved.

Basically, the two types of β-glucan molecules, which are based on the glycosidic bonds present in them, are α-glucan (dextran with 1,6, starch with 1,4 and 1,6 glycosidic bonds) and β-glucan (cellulose with 1,4, zymosan with 1,3, laminarin with 1,3- and 1,6, lichenin with 1,3 and 1,4 glycosidic bond). Because of the complex structure in β-glucans, they have superior ability to activate the immune response and act as biological response modifiers [27]. Certain characteristics of β-glucan, such as ability to function normally on immune system without over-activating them [28], ability to lower the elevated levels of cholesterol [29,30,31], and ability to reduce sugar levels [32,33], make it unique among immunostimulants.

β-Glucans are responsible for a multitude of actions, which protect and enhance the immune system and provide optimum resistance to any possible health assailants due to its ability to bind directly with macrophages and other white blood cells (neutrophils and natural killer (NK) cells) and activate them [34,35]. When β-glucan receptors are engaged by β-glucan, all immune functions are improved including phagocytosis (ability to engulf foreign cells and particles); release of certain cytokines such as IL-1, IL-6, GM-CSF, and interferons; and the processing of antigens. These cytokines stimulate formation of new white blood cells, providing immunity to β-glucan binding receptors present in all vertebrates ranging from fish to human [36].

## 4. Overview of Fish Immunity

Like cartilaginous fish, osteichthyes (commonly named ‘bony fish’) evolved through the Paleozoic Era, mainly during the Devonian. Whereas most early elasmobranchs and holocephalans are extinct, the bony fish continued to evolve in an expanding fashion. At present, the bony fish, in comparison to any vertebrate group, have reached the highest level of their evolution and adaptational capacities in an aqueous environment. Adaptationally, they may be considered as the most successful. Selectional pressures of various water environments, which the bony fish invaded, from fresh waters to tropical and polar seas, have induced a wide range of diversities in their body forms and size, so that they are also the most varied of all vertebrates [37,38,39]. Since the Tertiary Period, bony fish populations, especially the teleosteans (infraclass *Teleostei*), have increased, and currently form the most numerous group of all vertebrate taxa, nearly 35,000 species (i.e., comprising about one-half of all vertebrate species) [40].

The main cause of this unprecedented growth of various forms among the vertebrates was the emergence of new tissues and organs that enabled the realization of evolutionary novelty, the adaptive specific immunity with immunological memory. The first foundations of this type of defense of internal environment appeared in chondrichthyans (sharks, skates, and rays) together with the appearance of the jaws, which can be considered the most significant revolutionary event in the entire history of vertebrate evolution [41].

The immune system of bony fish is principally the same as in all advanced gnathostomeans. It is composed from two components, the phylogenetically older innate (nonspecific) immunity and the adaptive (specific) immunity with immunological memory. Contrary to higher vertebrates, survival of fish as the free-living organisms practically from the earliest embryonic stage depends mainly on their innate immunity [42], which remains the predominant component of defense throughout adult life.

The components of the innate immunity are divided into humoral molecules that are freely located in body fluids and cellular components. These include the growth inhibitors, lytic enzymes, agglutinins, precipitins (opsonins and primary lectins), cytokines, chemokines, and antibacterial (cationic) peptides [43,44], including components of complement (see below). This type of immunity is crucial in preventing infection due to slow proliferation and differentiation of immunocompetent cells after antigen stimulation, and limited antibody repertoire leading to a delay in the adaptive immune response especially in lower temperatures [45]. Therefore, the innate immune response acts as an alarm that allows the adaptive immune system time to mount a specific response [46]. The cellular components of innate immunity provide a physical barrier in the form of mucus-producing epithelial cells that line the skin and gills and specialized cells protecting the digestive tract, which are responsible for preventing penetration of pathogens inside the body. They comprise such cellular types like granulocytes, monocytes, macrophages, two types of NK cell homologues characteristic for fish, the non-specific cytotoxic cells and NK-like cells, and also the nonspecific cytotoxic cells, which kill and digest the pathogenic bacterial and viral invaders [47]. Further cellular components of innate immunity form various populations of blood leukocytes, which produce a row of abovementioned humoral substances that are immediately able to kill altered or foreign (allogeneic or xenogeneic) cells. To them, it is necessary to take into account the components of complement system, which, as seen in evolutionary advanced vertebrates, can be activated in three ways: the classical pathway (triggered by antibody binding to the cell surfaces); the alternative pathway (triggered independently of specific antibodies only by microorganisms); and the lectin pathway (triggered by the binding of a protein complex consisting of mannose/mannan-binding lectin in bacterial cells [43,48,49].

The adaptive immune system is well defined in mammals, and although most of the basic characteristics are also found in fish, the adaptive system is fairly inefficient due to a restricted antibody repertoire and an extensive lag time, up to 12 weeks after infection for activation [44,50]. In addition, due to their evolutionary status, cold-blooded vertebrates, such as fish, lack certain histologically distinct lymphoid architecture, such as follicular dendritic cells (DC) and germinal centers, which explains why fish are heavily reliant on a strong innate system [36,51,52,53].

It also plays a key role in the acquired immune response through a system of receptor proteins, which recognize pathogen-associated molecular patterns (PAMP), such as LPS and peptidoglycans, including bacterial and viral DNA and RNA.

The fish adaptive immune response takes place thanks to the presence of well-developed and functionally specialized structures and organs, like in higher gnathostomeans, the thymus, kidney, spleen and gut-associated lymphatic tissue (GALT), which form a sophisticated network of highly specialized cells, molecular messengers, and effective factors maintaining the homeostasis of an internal milieu [54].

The thymus together with the kidney (anterior and posterior) and spleen are the largest lymphoid organs in teleosteans [55]. Thymic structure, contrary to higher vertebrates, is highly variable and, in many species, is not possible to clearly differentiate between the cortex and the medulla [56].

The kidney in teleost fish is the equivalent of bone marrow in vertebrates and is the largest site of hematopoiesis until adulthood [55]. The main cells found in the anterior kidney are macrophages, which aggregate into so-called melanomacrophage centers, and lymphoid cells, which are found at all developmental stages (mainly B cells) [57].

The spleen is distinctly divided into white and red pulps, even if structurally less organized. This dividing is very variable. In some species, the red pulp prevails and may include the whole organ, whereas in others, it may be composed only of lymphoid cells and macrophages [58,59]. Fish spleen is a main site of erythropoiesis, phagocytosis, and antibody formation. The splenic tissue contains a system of ellipsoids and melanomacrophage centers. In most species, ellipsoids are clustered together and organized around the other two components [60]. The ellipsoids are thick-walled capillaries that open in the pulp and result from the division of the splenic arterioles. The macrophages along the capillaries are engaged on active phagocytosis of foreign material. A similar splenic structure has been described also in other teleosteans. The appearance of immunoglobulin-producing cells in the ellipsoids approximates them to true germinal centers of endothermic vertebrates; they could represent evolutionary predecessors of germinal centers of mammals [61]. In bony fish, it is generally accepted as a main secondary lymphoid organ, in which a plenty of B cells are activated and differentiate into plasma cells. Plasma cells then migrate to the other lymphoid organs such as head-kidney, intestine, skin, and gills. In the intestine, the distribution of B cells is low and variable among different species of fish [62].

In fish, the aggregations of lymphoid cells, plasmacytes, granulocytes, and macrophages present in connective tissue of the mucosa and infiltrating gut epithelia, including the lamina propria, represent, functionally, the GALT, but without organized structures resembling Peyer patches found in the mammals. These aggregates of immunocytes play the same role as an effective mammalian GALT. Together with the epithelial cells, these accumulations may form a microenvironment for food antigen collecting such as M cells [63]. Accumulations of lymphoid cells and cells producing antibodies have also been found in areas exposed to pathogens such as the skin epidermis, gills and pharynx, heart, liver, and pancreas. Generally, in the abovementioned organs and tissues, the plasma cells and lymphocytes are ultra-structurally similar to those of other ectotherms and endotherms.

Humoral factors are soluble proteins of the plasma and body fluids. These include transferrin, interferons, protease inhibitors (notably C3 and α2-Macroglobulin), lytic enzymes, proteins of the three complement pathways (classical, lytic and alternative), pentraxins, natural antibodies (NAbs), cecropins, and a whole host of cytokine and chemokine signaling messengers, notably IL-1β, TNFα, IL-2, IL-4, IL-6, IL-18, IFN1, IFN2, IFNγ, Th1, and possibly Th2 cytokines [43,44,64,65,66,67].

Bony fish are also the first vertebrates in which appeared genes of immunoglobulin superfamily molecules, the TCRα/β TCRγ/δ, β2-microglobulin, major histocompatibility complex (MHC) I class and MHC II class. Vβ, Dβ, Jβ, and Cβ regions are also present. It was suggested that fish TCR may be close in shape to the ancestral molecule [68]. Teleostean B-cells produce IgM (tetrameric), IgD, and IgT (also called IgZ) immunoglobulins but not IgA [69,70,71]. Immunoglobulins of fish are found in the skin, gut, gill mucus, bile, and systemically in the blood plasma [72].

Finally, it should be noted that physiological reactions, including immunological ones, are temperature-dependent in fish as in ectothermic animals, but are also affected by fluctuations in other external stressors such as salinity, photoperiodicity, oxygen concentration, pH changes, and especially by pollution.

Conversely, several food additives and modifiers of biological activity (especially with proven immunostimulants effects) [73], particularly β-glucans and also nucleotides [74,75,76,77,78], and probiotics [79,80,81] can enhance overall health of especially those fish species that are farmed in aquacultures.

### Mechanism of β-Glucan Action

β-Glucans have been proven to be highly efficient stimulators of the cellular and humoral branches in mammals and lately in other species, including invertebrates. The best-known effects of β-glucans consist of the augmentation of phagocytosis of granulocytes, macrophages, and DC. In this regard, macrophages are considered the basic effector cells in host defense. Most of the PAMP studied activate antigen-presenting cells together with native T cells into DC and T helper cells [82,83,84,85]. During microbial breakdown/degradation, numerous PAMP may be released initiating inflammatory responses upon receptor binding and intracellular activation of signal transducers and transcription factors.

The initial step of β-glucan–macrophage interaction is binding to specific receptors present on a cell membrane. In most animals, several receptors are involved in β-glucan recognition and binding: toll-like receptor 2 (TLR-2) [86], dectin-1 [87], CR3 (complement receptor 3, CD11b/CD18) [88,89,90], lactosylceramide [91], and less defined scavenger receptors. The binding has been confirmed not only by inhibition via specific antibodies, but also using KO mice [92].

The CR3 receptor, known also as Mac-1 or αMβ2-integrin, is highly promiscuous pattern-recognition receptors recognizing many other ligands, among them β-glucan.

CR3, known as membrane attack complex 1, is mainly expressed on myeloid cells, such as NK cells, DC, macrophages, monocytes and neutrophils, and functions as an eliminator to clear iC3b-opsonized doddery/apoptotic cells as well as pathogens [93]. CR3 is a dimeric integrin consisting of aMb2 (CD11b/CD18), two transmembrane proteins, and can recognize and bind to β-glucans through aM [94]. β2 is responsible for transmitting signal of aM to Syk pathway, resulting in CR3-mediated cytotoxicity (CR3-DCC) [95]. As the first observed β-glucan receptor, it is not surprising that most of our knowledge about β-glucan receptor interaction was gained here. The binding is complement-mediated and requires opsonization by iC3b, as confirmed by detection of iC3b and by direct binding (for review, see Bose et al. [88]). Details of the role of complement and β-glucan receptors in macrophage activation are summarized by Chan et al. [96].

**Figure 1:**
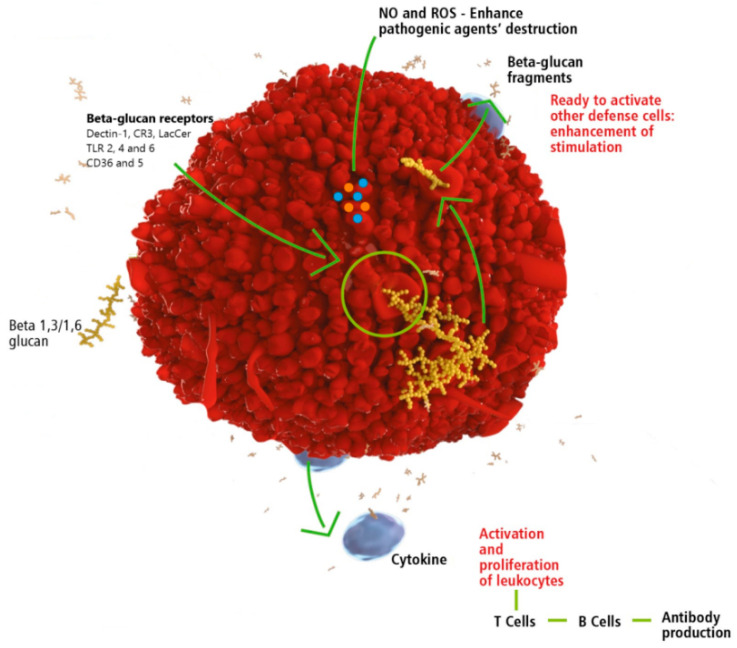
Illustration of the general mode of action of β-1,3/1,6-glucan on leukocytes (neutrophils, monocytes, natural killer cells, or macrophages). The β-glucan receptors may change according to the vertebrate species and leukocyte type. Figure kindly provided by Biorigin.

After establishing CR3 as the primary β-glucan receptor, dectin-1 was recognized as another major β-glucan receptor, present on numerous cell types. Using specific anti-dectin-1 antibodies, several studies found that this receptor is almost exclusively responsible for binding of β-glucan and zymosan [97]. Dectin-1 receptor was also shown to be involved in recognition of pathogenic fungi and in secretion of IL-12. Experiments using antifungal response of NK cells showed that dectin-1 response to β-glucan binding starts IL-12 production by antigen-presenting cells with subsequent trigger of NK cells to start IFN-γ production [98]. Major receptors are shown in Figure 1.

Recently, the focus switched from dectin-1 and CR3 receptors to TLRs, which are receptors with important roles in innate immunity. Curdlan (water-insoluble linear beta-1,3-glucan consisting of β-(1,3)-linked glucose residues and forms elastic gel upon heating in aqueous suspension) was found to act on various cell types via binding to TLR-2. This binding was acting through suppression of expression of RANKL [99].

Although the mechanisms are unclear, the interaction among β-glucan and receptors might depend on factors such as solubility, as only insoluble β-glucans cluster dectin-1 receptors with subsequent expulsion of negative regulators such as CD148 or CD45 [100]. β-Glucan has been found to active microglia via dectin-1, but the same group later described that nonsoluble β-glucan acted via TLR-2 and TLR-4 and stimulated reactions, which were unaffected via dectin-1 [101]. The action via TLRs is probably mediated by suppression of NF-κB activation.

Dectin-1 is a well-researched C-type lectin receptor (CLR) that is responsible for β-glucan recognition and plays an important role in antifungal infection [102]. It recognizes β (1,3) and β (1,6) linked β-glucans and the binding strength depends on the size, linkage type, and branching degrees of the β-glucans [103,104]. This receptor can mediate the activation signal to enhance an immune response and is called a β-glucan receptor. It is expressed on numerous cell types including DC, macrophages, monocytes, neutrophils, and T cells. It mainly exists on cell passageways where pathogens can easily invade and can mediate pathogen recognition and phagocytosis, which play an important role in host defense [105]. The activation of dectin-1 will also result in DC maturation, ligand phagocytosis, respiratory burst, and arachidonic acid metabolization for host defensive immunity [106,107,108].

To further complicate the situation, some β-glucan can bind to dectin-1 in combination to extracellular TLR [108]. This process might first involve activation of dectin-1 and subsequent complexation of TLR, as described with TLR-2, TLR-6 [34], and TLR-4 [109]. A detailed study using cells coexpressing dectin-1 with TLR-2, TLR-4, or TLR-5 found not only the differences in activity of soluble and nonsoluble β-glucans, but also that the immune effects of β-glucan differed based on the dectin-1/TLR combination [110]. This might explain at least some of the differences in β-glucan activities. Even less clear claims were raised by Su et al. [111], who found that a (1-6)-(1,4)-β-d-glucan inhibits cytokine production and that this activity is mediated via binding to TLR2 but not to dectin-1 or CR3 receptors. This study is confusing not only due to the rather unusual binding patterns, but also due to the first description of inhibition of cytokine synthesis by β-glucan. So far, all β-glucans either stimulated cytokine production or, as seen with betafectin, had no effects.

Latest experiments suggested the role of programmed cell death protein 1 (PD-1) immuno-checkpoints and the involvement of c-Maf. Treatment with β-glucan reduced c-Maf expression in M2 macrophages together with reduction of some populations of monocytes. In clinical trials, the same treatment decreased the numbers of inflammatory monocytes and increased the numbers of classical “patrolling” monocytes responsible for regulation of tumor metastases. These data suggest the possible benefits of targeting immunosuppressive macrophages and offer a new look at possible mechanisms of β-glucan action [112]. The pathways in which β-glucans mediate their activity in fish are not fully elucidated but, as expected by the well-conserved innate system, so far appear similar to that of mammals.

Complement protein C3 and lectins (possibly dectin-1 homologues or similar) have been identified as β-glucan pattern recognition receptors, as well as a β-glucan pattern recognition receptors on salmon macrophages and catfish neutrophils [43]. In a model of regulation of a gene expression profile, the typical signaling pathway associated with CLR activation and the identification of several candidate β-glucan receptors suggested that immunomodulatory effects of β-glucan in carp macrophages could be a result of signaling mediated by a member of the CLR family [113]. TLR homologues have also been described in Atlantic salmon, Zebrafish, flounder, goldfish, and pufferfish [44].

However, the situation in fish is less clear. So far, no clear homologue of dectin-1 has been found. A detailed study of β-glucan recognition by fish cells suggested possible receptors belonging to the CLR family [113]. An analysis of the carp genome found 239 genes encoding proteins with some C-type lectin domains, but even after additional analysis, no receptor was found on macrophages. Therefore, even when CLR family is the most promising β-glucan-binding moiety, the exact mechanisms of β-glucan recognition in fish are still unclear. However, effects of curdlan, β-glucan known to bind to dectin-1, suggest the presence of a similar binding side. Detailed studies found several candidates with similar protein architecture. Subsequent mining of the zebrafish genome revealed two genes as candidate β-glucan receptors [113]. With respect to the CR3 receptor, there is only indirect proof of the existence of this receptor in fish.

## 5. Routes of β-Glucan Administration

β-Glucans can be administered internally and externally in a number of different routes such as intravenous, intraperitoneal, or subcutaneous (parenteral) injections; orally; bathing; or as part of a cream [114,115,116]. Efficacies of different routes (intraperitoneal injection, bathing, and oral administration) have been tested. In a model with *Cyprinus carpio*, fish were fed with β-glucan and LPS to investigate survival and immune response after challenged with *Aeromonas hydrophila*. Intraperitoneal injection showed 100% relative percentage survival at all concentrations of β-glucan, whereas oral administration showed high relative percentage survival at higher concentrations (1% β-glucan + 0.25% LPS), but bathing did not improve relative percentage survival levels [117]. In a model of streptococcus caused by *Streptococcus iniae*, a formalin-killed vaccine was applied in red tilapia by injection, immersion, and oral vaccination. The result from the study indicated that the best route regarding efficacy was through intraperitoneal injection and that soluble β-glucan increased further the effectiveness of the vaccine [118].

### 5.1. Injection

The protective effect of β-glucan injection in a dose-dependent response has been demonstrated in different species against several infections [119,120,121]. The intraperitoneal injection certainly is an effective method to deliver β-glucan and stimulate the immune system but is not the most practical method. For example, a single dose of β-glucan injected intraperitoneal in rainbow trout resulted in a level of protection against infection with the microsporidian, *Loma salmonae*, similar to the level of protection induced by a 3-week feeding trial using 10 times higher concentrations of β-glucan. Interestingly, the effects of the single intraperitoneal injection could be measured for a prolonged period of up to 9 weeks in vivo [122] and up to 20 days ex vivo (no further time points measured) [123]. This concept may explain the application of β-glucan as a vaccine adjuvant. Glucan does not need to be a direct part of the vaccine, it can serve as an important supplement, as shown by experiments that found significant enhancement of the immersion efficacy on inactivated herpesvirus vaccine in Gibel carp [124].

Phagocytes could be responsible for long-lived effects induced by β-glucans since intraperitoneal injection with β-glucan leads to an increase in oxidative burst, phagocytosis, and lysozyme activity of macrophages in Atlantic salmon [123]. In addition to this, another study showed that increased macrophage activity was still measurable at 10–20 days post-injection, providing clear indications that single intraperitoneal injections with β-glucans can induce long-lived effects in fish [125].

Due to this immunomodulator action, β-glucans have been extensively studied as vaccine adjuvants or as vaccine delivery systems [126,127,128]. In a model with *Vibrio damsela* vaccine, turbot (*Scophthalmus maximus* L.) were injected prior, together, and post-application of yeast β-glucan. The highest activity among all the immune parameters was obtained when β-glucans were injected after the bacterin application. The finding of this study indicates that the sequence of β-glucan administration is critical in order to use β-glucan as a vaccine adjuvant [129].

Apart from the studies about adjuvant activity of β-glucan in vaccine formulation directly, pattern antigens such as ovalbumin (OVA) or bovine serum albumin (BSA) are commonly combined to investigate the adjuvanticity and mechanism of β-glucans. When microparticulate β-glucan (MG) was covalently conjugated to OVA or BSA and administered to animals, the MG-antigen complex was phagocytosed by DC or macrophages via specific receptors that recognize β-glucan, then resided in vesicles and presented by MHC II to activate CD4^+^ T cells or by MHC I to activate CD8^+^ T cells through cross-presenting, and the expression of costimulatory molecules, such as CD25, CD69, and B7, were upregulated to strengthen the activation signals [130].

### 5.2. Dietary

Orally delivered β-glucans make their way to the gastrointestinal tract, where they must first be captured into the circulation before being conducted the bone marrow. The linear β (1,3) backbone ends up undigested in the proximal part of the intestine, where a proportion is captured by M cells in cooperation with neutrophilic granulocytes or macrophages and degraded by the latter under a reactive oxygen species-driven process [131]. Despite the low systemic blood levels of β-glucans (less than 0.5%), significant systemic immunomodulating effects in terms of humoral and cellular immune responses were demonstrated [96]. In addition, the higher part of the non-digestible β-glucans may induce alterations in the composition of the gut microbiota, thereby indirectly influence the local immune system or the bacterial community in the gut. These effects are most probably manifested through decreasing *Firmicutes* and increasing *Akkermansia* populations. This bacterial community may help to digest non-digestible oligosaccharides, such as β-glucans, into short-chain fatty acids with a physiological effect of their own [132]. Taken together, these different paths can help explain part of the previously described effects of β-glucans. In short, in promoting hepatic glycogen synthesis through improving IRS/Akt insulin signaling pathway, inhibiting the sodium-glucose linked transporter 1 (SGLT-1) expression in intestines and decreasing blood glucose, suppressing macrophage infiltration in adipose tissues, and decreasing of TNF-α in blood and muscles [133]. A seven-week supplementation of carp (*Cyprinus carpio*) with β-glucan did not stimulate expression of bactericidal innate immune genes but changed bacterial composition in the gut [134]. Some studies suggested that the effects of β-glucan might not be strong enough to elevate the immune response of fish. However, the simultaneous use of oxytetracycline with β-glucan supplementation ameliorated the immunosuppressive effects of antibiotics and help protect fish against bacterial infections [135]. The stimulation resulting from β-glucan supplementation is dependent on numerous factors including dose, time of supplementation, water temperature, and species [136]. In vitro experiments showed the high doses of β-glucan inducted apoptosis in primary cells isolated from carp pronephros, but the doses routinely used in aquaculture do not induce apoptosis, but stimulate immune system [136]. The short-term feeding with β-glucan did not change expression of immune genes in striped catfish (*Pangasianodon hypophthalmus*), but after subsequent challenge with *Edwardsiella* infection, the β-glucan-supplemented group showed significant stimulation of immune genes in all tested organs [137]. These results suggest that the effects of β-glucan feeding in healthy adult fish are minimum, but this feeding has immediate effects even 24 h after infection. Similar results were found in case of juvenile pompano (*Trachinotus ovatus*), where the effects of β-glucan feeding to healthy individuals were small, but after infection with *Streptococcus iniae*, β-glucan offered significant protection [138]. A model of silver catfish (*Rhamdia quelen*) and *Aeromonas hydrophila* infection offered similar results [139]. In general, these studies demonstrated a lack of β-glucan effects when fish are in resting and a significant and positive effect when fish are exposed to a disturbance in the homeostasis, usually by stimuli such as stress [140], immunological challenges by pathogens [137,138] or chemicals (“immunocompromised”) [141]. Therefore, authors should consider this point to discuss the lack of β-glucan effect in resting.

The orally delivered pathway is much slower and said to have a less profound effect than injectable methods. However, this is often a more practical method as β-glucans can simply be added to food/feed [94,115,141,142,143]. For example, Rodriguez et al. [140] fed salmon a diet supplemented with β-glucan and found that the β-glucan diet potentiated the immune response to vaccine by increasing innate and adaptive immune responses through the transcription of key cytokine genes such as INF-γ and IL-12.

In sea bass fed with β-glucans for 4 or 8 weeks, pyrosequencing of the intestinal microbiota revealed a transient alteration at the family taxonomic level in the composition of the autochthonous microbiota [144]. It took a period of 4 weeks to completely shift the dominance within the microbial communities, which returned to the original composition after another 4 weeks of feeding. The data presented in these studies imply that effects of oral administration of β-glucans on the microbial composition in the gut are present but could be transient and require further investigation. In line with these findings, the effect of long-term feeding with β-glucans on TLR3 expression in the gut of carp could also be due to an indirect effect of β-glucans on the composition of the microbiota [125].

Studies investigating the effects of β-glucans on maintaining the integrity of the gut have found no adverse effects and provide evidence for an assumed favorable increase in frequency of mucus-secreting cells in the epithelial barrier [145,146]. Approaching this subject, oral administration of β-glucans to rainbow trout appears to downregulate the expression of immunoregulatory genes (e.g., IL-1β and lysozyme) in the presence of a microbial stimulus [147,148], but upregulate the expression of such genes (e.g., IL-1β and cathelicidins) in the absence of a microbial stimulus [146,148]. These apparent contrasting effects of β-glucans on the expression of immunoregulatory genes, in the presence or absence of a microbial stimulus, could possibly help explain the variable outcomes with respect to increased resistance against pathogens [125].

In most of the studies performed on bass species, oral administration of β-glucans not only increased innate immune parameters, such as phagocytic capacity and oxidative burst, lysozyme and complement activity [149,150,151,152], but also protected against challenge with numerous bacterial pathogens including *Aeromonas hydrophila* and *Vibrio alginolyticus* [149,151].

Bagni et al. [153] reported duration-dependent effect of dietary application where significant elevation of serum complement activity in sea bass fed with β-glucans at 15 days was seen; however, serum lysozyme, gill, and liver heat shock protein concentrations were enhanced at 30 days. Long-term use had no significant impact on innate and specific immune parameters, survival, growth performances, and conversion index in treated and control fish. Continuous feeding with β-glucans for a number of subsequent days also appears to induce long-lived effects on the immune system of fish. For example, rainbow trout fed with β-glucans for a period of 2 weeks still showed higher antibody responses after vaccination against enteric redmouth disease and higher concanavalin A-induced proliferation of head kidney derived leukocytes 4 weeks after switching back to a control diet [154]. Grouper fed a diet containing a mixture of mushroom-derived β (1,4) (1,3) and β (1,6) glucan for a continuous period of 12 days still showed higher protection against challenge with *Vibrio alginolyticus* 15 days after switching back to a control diet [149].

Continuous administration of β-glucans generally appears to result in an increased expression of pro-inflammatory genes, with a gradual decline over time depending on, among others, route of administration and immune organ under investigation [155,156]. Oral administration (25 days) of β-glucans can result in the upregulation of anti-apoptotic genes in gut and head kidney, and of both anti- and pro-apoptotic genes in the spleen of common carp [157]. The effects of β-glucans on apoptosis were further investigated and show that, in vitro, β-glucans can have a significant effect on apoptosis, but only at very high concentrations [136]. Taken together, these findings support the notion that oral administration of β-glucans may modulate the intestinal immune response and protect cyprinid fish from an acute (over)reaction [155,158].

In an in vitro study, head-kidney macrophages of pink snapper (*Pagrus auratus*) pre-incubated with commercial β-glucan (EcoActiva) and subsequently exposed either by phorbol myristate acetate (PMA) or LPS resulted in significant stimulation of superoxide anions and respiratory burst activity compared to induction of macrophage with EcoActiva alone [159]. The result of this study demonstrates that feeding of β-glucan may enhance the recognition of LPS present in the cell wall of Gram-negative fish pathogenic bacteria resulting in improved killing efficiency of macrophages of these pathogens [160]. In another study, oral administration of EcoActiva in pink snapper increased macrophage O_2_ radicals especially in wintertime, but no enhancement in classical and alternative pathway activities was seen, indicating wintertime to be the most favorable to feed snappers for disease resistance [161].

Yeast β (1,3)(1,6) glucans have been used for in vitro and in vivo experiments to study degranulation of primary granules in fish neutrophils [162]. β-Glucan supplied to nonstress (NS), acute stress (AS), and chronically stressed (CS) fish showed increase degranulation in NS and prevented decrease of degranulation in AS, whereas in CS fish, degranulation reached NS level after 3 days of feeding in fathead minnows (*Pimephales promelas*, *Rafinsesque*). These results indicate that β-glucan supplementation to fish diet prior to AS and during CS can enhance neutrophils function and increase disease resistance and survival rate [154].

Large yellow croaker were fed with diets supplemented with 0% (control), 0.09% (low), and 0.18% (high) of β (1,3) glucan for 8 weeks; the results showed low β-glucan levels enhanced fish growth, while high levels significantly enhanced the lysozyme activity. Respiratory burst activity in head-kidney macrophages was enhanced with low concentration of β-glucan. Overall growth, lysozyme, phagocytosis, respiratory burst, and protection against *Vibrio harveyi* were enhanced but there was no effect on alternative complement pathway [163].

Dietary supplementation of aflatoxin (AFB1) in fish showed reduced immunity with affected biochemical parameters related to organ damage. Nile tilapia immunocompromised with aflatoxin (200 Lg/feed aflatoxin B1) and fed for 21 days with diet supplemented with 0.5% of β (1,3) glucan showed enhanced resistance against *Streptococcus iniae* and improved non-specific immunity levels compared to AFB1 non-treated fish. Superoxide anion, myeloperoxidase, phagocytic activity, and hemagglutination were also increased [164,165], and the authors concluded that the use of β (1,3) glucan as feed supplement resulted in enhanced immune response in immunocompromised fish.

Cyclophosphamide, a multifunctional alkylating agent, as well as a cytotoxic drug and a well-known immunosuppressant, was used to induce an immunocompromised state in Asian catfish (*Clarias batrachus*) [164,165]. The cyclophosphamide-treated fish showed lowered levels of respiratory burst, myeloperoxidase, and phagocytic activities in blood phagocytes, and decreased hemagglutination activity. β-Glucan delivered as feed supplement significantly enhanced these immune parameters. Taken together, the use of β-glucan may have advantages during immunosuppressive states, such as during physiological and environmental stress. The feed manufacturers are also advising to use feed with immunostimulants during such circumstances [166].

Overall, it is becoming clear that oral administration of β-glucans stimulates the innate immune system of cyprinids as it stimulates the innate immune system of salmonid and perciform fish species, suggesting that the capacity to stimulate the innate immune system of fish is a capacity intrinsic to (large molecular weight) β-glucans [125].

Kock et al. (manuscript in preparation) fed Nile tilapia for 0, 15, 30, or 45 days with a diet containing 0.1% of β-glucan (MacroGard), and evaluated the growth performance at the end of the feeding trial, and the innate immune function immediately after the feeding trial, and 7 and 14 days post-treatment (i.e., withdrawal period). The authors found that independent of the administration periods, fish fed with β-glucan had relatively higher innate immune responses, such as lysozyme activity in plasma, liver, and intestine and respiratory burst, compared to control and, overall, these differences became smaller over the withdrawal period. Moreover, at day 10 post-treatment, fish were challenged with bacteria (*Aeromonas hydrophila*); the control group had early mortalities (2 vs. 4–5 days post-infection, respectively) and lower survival rate (60% vs. 80%, respectively) compared to fish fed with β-glucan for 15 or 30 days, and, interestingly, fish fed for 45 days with β-glucan had no mortality. This study indicates that independent of the administration periods (i.e., 15 to 45 days), the β-glucan improved the innate immune responses and tilapia resistance to disease, and this protection could be observed up to 10 days post-treatment. The most relevant is that long-term administration did not cause immunosuppression as previously hypothesized due to an exhaustion of the immune system, but surprisingly promoted an even better growth and immune performance.

Regarding the period of administration, some studies have proposed that the longer administration may cause an overstimulation or a distress generated by the high energy cost due to prolonged exposure to β-glucan [21,167,168,169]. However, none of the studies that compared periods of β-glucan administration [149,153,170,171,172,173,174,175] found evidence that longer administration periods (up to 56 days) negatively impact the immune system. The studies that reported a negative effect used a high dietary inclusion level (e.g., >0.1%) or injected fish. These treatment protocols may have led to an exacerbated/toxic amount of β-glucan [163,167,174,176]. Taken together, these findings indicate that longer administrations periods (i.e., >4 weeks) can be beneficial at a low dose, reinforcing the hypothesis suggested by Ai, Mai, Zhang, Tan, Zhang, Xu and Li [163], Douxfils, Fierro-Castro, Mandiki, Emile, Tort and Kestemont [174], and Do Huu, Sang and Thanh Thuy [175] that immunosuppression may be caused by high dose.

### 5.3. Bath

A potentially interesting alternative application of immunostimulation induced by β-glucans is provided by the immersion treatment. For example, a short β-glucan bath of 3 min in fertilized eggs or gametes of chum salmon (*Oncorhynchus keta*) was sufficient to provide significant protection against infection with *Saprolegnia* spp., [177]. This finding was supported by the observation that both pro- and anti-inflammatory genes were upregulated after immersion of rainbow trout fry in a solution containing β-glucan [178].

It is essential to know the correct dosages of immunostimulants and appropriate administration route to achieve the desired results. Chinook salmon were fed with a diet containing 0%, 0.01%, 0.1%, and 1.0% of β-glucan for 7 days or immersion administration of β-glucan, and thereafter fish were bath challenged with *Aeromonas salmonicida*. Diet containing 0.1% and 1.0% of VitaStim-Taito glucan resulted in significant protection against *A. salmonicida*, but no significant protection was noted in any of the group bath treated [179].

Administration of β-glucans by immersion, as modulators of mucosal surfaces of the skin or gills, could be a promising new area of research, especially now that tools to reliably measure mucosal immunity are becoming available [180]. Possible explanations for immunostimulating effects of β-glucan immersion baths could be sought, for example, in effects on the composition of microbial communities in the skin mucus [181] or increased local populations of alternatively activated macrophages expressing a healing phenotype [182].

A summary of β-glucan effects on fish is shown in Table 1.

## 6. New Insights about the Use of β-Glucan in Aquaculture

β-glucan seems to affect more physiological conditions than “only” the immune system. A study of rainbow trout (*Oncorhynchus mykiss*) given a food supplement for 60 days and using proteomic analysis, found changed expression of structural muscle proteins. The authors speculate that these alterations might be responsible for improved growth rate in fish [212].

Among direct effects on improvement of various immune reactions, β-glucan supplementation can have additional nutritional effects including amelioration of toxic effects caused by deltamethrin. Experiments using Nile tilapia showed improved cortisol levels and significantly reversed inflammatory and transcriptomic damages caused by the toxin [213]. In addition, β-glucan feeding ameliorate cold stress-related mortality in *Pangasianodon hypophthalmus* [214], but glucose and cortisol levels remained unchanged. Environmental stress caused by either overcrowding or by environmental pollution, is one of the problems the current aquaculture suffers from. Food supplementation with β-glucan was found to improve ammonia-related stress in *Oreochromis mossambicus* via improvements of cellular, humoral, and antioxidant response [215].

**Figure 2:**
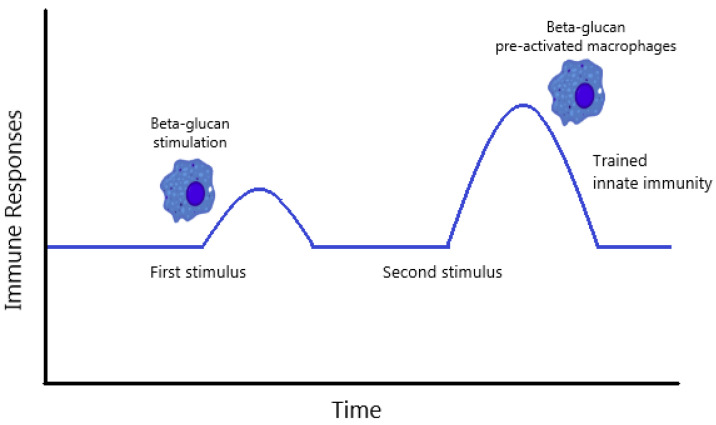
The concept of “trained innate immunity” adapted from Alvarez-Errico et al. [217] and Petit and Wiegertjes [125].

Recent insights in the field of innate immunity provide indications that β-glucans could also have effects for a longer period, possibly explained by the phenomenon of ‘trained immunity’ [125]. At present, the strict absence of a form of memory for innate immune responses is challenged by a new concept named trained immunity, which is characterized by three criteria: (i) it can be induced after a primary infection or immunization and subsequently provide protection against a secondary infection in a T- and B-lymphocyte independent manner; (ii) it may be less specific than the adaptive immune response but still confers increased resistance upon reinfection of the host; and (iii) innate cell types, such as macrophages and NK cells, are key players in the mechanism, which involves improved pathogen recognition and an increased inflammatory response [216]. A concept of trained immunity is shown in Figure 2.

Another possibility of glucan action is the potential effect on neuroendocrine axis. It is well established that the neuroendocrine and immune systems communicate bidirectionally via numerous cytokines acting as auto/paracrine or endocrine factors regulating pituitary development, cell proliferation, hormone secretion, and feedback control of the hypothalamic-pituitary-adrenal axis. However, the information on these possible effects of glucan in fish is still lacking.

Effects induced by vaccination with Bacille Calmette-Guerin (BCG) [218,219], prepared from attenuated live *Mycobacterium bovis*, support the proposed benchmarks of trained immunity that it can elicit cross-specific protection in a T- and B-cell independent manner with innate immune cell types, such as macrophages, acting as key players [216]. Of evolutionary interest, long before the recent discussions on the presence of trained immunity in humans and mice [220], similar cross-specific protection was observed in plants [221,222,223] and invertebrates [224], which, typically without T and B lymphocytes, can build up a form of immunity to protect the organism from a secondary exposure. Owing to the basal position of teleost fish as early vertebrates, it makes evolutionary sense to expect that trained immunity could be an important mechanism determining immunostimulation of fish by β-glucans [125].

There are a few studies providing evidence for the presence of a form of trained immunity in fish, primarily based on experiments with mycobacteria. Olivier et al. [225] observed a long-lived increase in phagocytic activity of peritoneal macrophages from brook trout (*Salvelinus fontinalis*), for a period up to 33 days after intraperitoneal injection with modified Freund complete adjuvant containing killed *Mycobacterium butyricum*. Only macrophages from trout injected with modified Freund complete adjuvant showed a significantly higher bactericidal activity

Vaccination of Japanese flounder (*Paralichthys olivaceus*) with BCG resulted in an upregulation of pro-inflammatory cytokines and conferred protection against *Mycobacterium* sp. [226]. Moreover, vaccination of Amberjack (*Seriola dumerili*) with BCG led to protection against challenge with *Mycobacterium* sp. [226]. Importantly, these researchers could measure cross-specific protection, one of the proposed benchmarks of trained immunity. The cross-specific protection could be induced in Japanese flounder by BCG, shown by challenge with *Nocardia seriolae*, and was possibly mediated by bacteriolytic activity of the serum [227].

Cross-specific protection occurring in a T- and B-cell independent manner [216] was also studied in fish. Exposure of Rag-KO zebrafish to a sublethal infection with *Edwardsiella ictaluri* significantly protected the same animals from a subsequent lethal infection with the same bacteria. Importantly, protection could be transferred to native Rag-KO individuals by injection with kidney leukocytes from animals pre-exposed to the sublethal infection [228].

According to Petit and Wiegertjes [125], it remains to be investigated if trained immunity has the predicted, pronounced role in the immune defense of fish, and is indeed mediated by innate immune cell types, such as macrophages.

## 7. Conclusions

Currently, more than 3000 papers have reported the effect of β-glucan on immune responses in fish; however, several questions remain. Detailed knowledge of the receptors involved in recognition of β-glucans and of their downstream signaling is missing for teleosts, leaving obscure whether the observed potentiation should be attributed to direct effects on leukocytes or to indirect effects on, for example, the composition of microbial communities in the gut. Typically, studies investigating the effects of β-glucans have mostly focused on relatively short-lived effects, in the order of days up to a few weeks, but recent insights in the field of innate immunity provide indications that β-glucans could also have effects for a longer period of time, possibly explained by the phenomenon ‘trained immunity’.

## Figures and Tables

**Table 1:** Major effects of feeding with β-glucan.

Species	Dose	Trial Duration	Main Effects	Reference
Atlantic salmon (*Salmo salar*)	500 or 1000 mg/kg diet	70 days	MacroGard reduced the number of lice-infested fish by 28%.	Refstie, et al. [183]
Red tilapia *(Oreochromis niloticus x O. mossambicus)*	Vaccine with adjuvant, the vaccine was emulsified in an equal volume of 2%	28 days	MacroGard increased the effectiveness of vaccine produced from *Streptococcus iniae* in fish.	Suanyuk and Itsaro [118]
Common carp (*Cyprinus carpio*)	10 mg/kg body weight	14 days	β-Glucan feeding did show significant effects on both CRP and complement profiles, suggesting that MacroGard stimulated CRP and complement responses to *A. salmonicida* infection in common carp.	Pionnier, Falco, Miest, Frost, Irnazarow, Shrive and Hoole [156]
Persian sturgeon (*Acipenser persicus)*	0.1, 0.2, or 0.3%	6 weeks	Lysozyme activity and ACH50 were significantly higher in 0.2% and 0.3% β-glucan fed fish. Elevated growth performance (final weight, specific growth rate, and food conversion ratio) was observed in fish fed 0.1; 0.2, or 0.3% β-glucan compared to the control group.	Aramli, Kamangar and Nazari [25]
Pompano fish (*Trachinotus ovatus)*	0, 0.5, 1, 2, or 4 g/kg diet	8 weeks	β-Glucan supplementation is effective for improving growth, intestinal *Vibrio* counts. Fish fed 0.05% or 0.20% β-glucan showed better resistance against salinity.	Do Huu, Sang and Thanh Thuy [175]
Common carp (*Cyprinus carpio*)	100 μg/mL (in vitro)	Not mentioned	β-Glucans stimulate carp macrophages to increase the production of reactive oxygen and nitrogen radicals and affect the expression patterns of cytokine genes that can differ among activated pattern recognition receptors.	Pietretti, et al. [184]
Atlantic salmon (*Salmo salar*)	0.1%	35 days	Results showed that the tested β-1,3/1,6-glucan diets increased the levels of transcripts of key genes involved in innate and adaptive immune response of salmon, potentiating the response to a model vaccine and also antagonizing the effects of hypoxia	Rodriguez, Valenzuela, Farias, Sandino and Imarai [140]
Nile tilapia (*Oreochromis niloticus*)	0.1% β-glucan + 600 mg vitamin C	7, 15, 30, or 45 days before challenge	Diet supplemented with 0.1% of β-glucan and 600 mg of vitamin C/kg fed for at least 15 days is recommended especially when fish are likely to encounter transport-induced stress.	Barros, et al. [185]
Nile tilapia (*Oreochromis niloticus*)	0.1% of each glucan	30 days	Different β-glucan samples exhibited biologically differently behaviors, but both increased the resistance against bacterial infection. Specifically, BG01 increased immunostimulation, while BG02 improved growth performance.	Pilarski, Ferreira de Oliveira, Darpossolo de Souza and Zanuzzo [11]
Turbot (*Scophthalmus maximus*)	0.5 g/L MacroGard (Artemia enrichment)	13 days post hatching	Mortality was significantly reduced by 15% and an alteration of the larval microbiota was observed. At 11 DPH, gene expression of trypsin and chymotrypsin was elevated in the MacroGard fed fish, which resulted in heightened tryptic enzyme activity. MacroGard induced an immunomodulatory response and could be used as an effective measure to increase survival in rearing of turbot.	Miest, et al. [186]
Matrinxa (*Brycon amazonicus*)	0.1% β-glucan	15 days	β-Glucan modulated the cortisol profile prior to and after the stressor, increasing the number and activity of leukocytes. Our results suggest that β-glucan-induced cortisol increase is one important mechanism to improve the innate immune response in matrinxa.	Montoya, et al. [187]
Nile tilapia (*Oreochromis niloticus*)	0.1, 0.2, 0.4, or 0.8% and vitamin C (400 or 600 mg/kg diet)	60 days	0.1–0.2% β-Glucan and 600 mg/kg vitamin C increased fish resistance to stress.	Barros, et al. [188]
Nile tilapia (*Oreochromis niloticus*)	0.1 or 0.2% of β-1,3/1,6-Glucans	21 successive days prior to bacterial challenge and during the seven days of sampling	β-Glucan can modulate the antioxidant, inflammation, stress, and immune-related genes in Nile tilapia, moreover, 0.2% β-glucans showed better protective effect with *Streptococcus iniae* challenge.	Salah, et al. [189]
Carp (*Cyprinus carpio*)	10 g MacroGard kg-1 diet	14 days prior bacterial application	In β-glucan fed carp, mucus was quickly released from the intestinal goblet cells and was probably washed out of the gut together with a high number of intestinal bacteria. This could indicate a form of protection against bacteria.	Jung-Schroers, et al. [190]
Atlantic salmon (*Salmo salar*)	15 mg/kg of fish (intubated fishes)	Not mentioned	This study provides some clues on the mechanisms by which the β-glucan evokes response in the fish, at the intestinal level.	Kiron, et al. [191]
Carp (*Cyprinus carpio*)	1% of feed	14	β-Glucan can boost the host innate immune defense by inducing neutrophil extracellular trap formation and by stabilizing neutrophil extracellular traps against bacterial nuclease degradation, and thereby reduce the severity of an infection of *A. hydrophila*.	Brogden, et al. [192]
Carp (*Cyprinus carpio*)	20 mg/mL in in vitro head-kidney cells	Not mentioned	β-Glucan stimulation of scratch-wounded fibroblasts cultures did not enhance wound recovery.	Vera-Jimenez and Nielsen [193]
Carp (*Cyprinus carpio*)	20 mg/mL in in vitro head-kidney cells	Not mentioned	Both methods compared during this study, showed the capacity to detect and measure the respiratory burst response of carp head kidney cells after stimulation with β-glucans.	Vera-Jimenez, et al. [194]
Rainbow trout (*Oncorhynchus mykiss*)	0; 0.1; 0.2; 0.5% of feed	15 × 30 days	Feeding low doses of β-glucans may help to boost immune function in case of a bacterial infection, especially the inflammatory response, while feeding high doses of β-glucans may result in a more or less rapid stress and immune exhaustion or feedback regulation, making appropriate response to subsequent pathogenic threat impossible. Additionally, the effects of β-glucans on the immune-related gene expression mainly concern spleen tissue, both prior and after bacterial infection, suggesting a targeted reinforcement of immune functions in this organ.	Douxfils, Fierro-Castro, Mandiki, Emile, Tort and Kestemont [174]
Matrinxã (*Brycon amazonicus*)	0.1% on feed	15	Inclusion of β-glucan in fish diet may help to prepare them to face stressful practices in fish farming.	Montoya, et al. [195]
Carp (*Cyprinus carpio*)	0.1; 1.0; 2.0% of feed	14 and 28	Dietary MacroGard may affect the composition of the carp intestinal microbial communities. Furthermore, positive effects on intestinal microvilli length and density were also observed. Indeed, these changes at 1% and 2% MacroGard supplementation might be contributory factors to the improved growth performance recently observed in carp fed 1% and 2% dietary MacroGard.	Kuhlwein, et al. [196]
Juvenile Pompano (*Trachinotus ovatus*)	0.1; 0.2% of feed	21 + 10 challenge	Supplementation of β-glucan in the diet is beneficial in boosting nonspecific immunity, growth performance, survival rate, and tolerance to *Streptococcus iniae* infection of pompano *T. ovatus*. The addition of 0.10% of β-glucan to the pompano diet is recommended to boost disease resistance, immunity, and growth performance.	Do-Huu, Nguyen and Tran [138]
Juvenile pompano (*Trachinotus ovatus*)	0; 0.05; 0.1; 0.2; 0.4; 0.5% of feed	56	The results of the present study confirmed that supplementation of β-glucan in the diet could improve the growth, protein content in flesh, feed conversion ratio, feed conversion efficiency, protein efficient ratio, and protein productive value in pompano, *T. ovatus*. It is recommended that supplementation of 0.5–1.0 g/kg β-glucan in the diet to obtain maximal growth, feed utilization and protein utilization of juvenile pompano.	Do-Huu, et al. [197]
Carp (*Cyprinus carpio*)	0.1% in vivo	42 days. Fish were sampled every week from week 2 to 6.	Application of MacroGard after the third week post hatching resulted in a significant increase in classical complement activity when compared to fish fed the control diet. The results demonstrate that feeding with β-glucan enriched diet enhances the immune defense parameters of juvenile carp.	Sych, et al. [198]
Carp (*Cyprinus carpio*)	6 mg/kg in vivo	14 days	β-Glucan supplemented diet administered to common carp decreased the transcript levels of several pro-inflammatory cytokines in gut and head kidney tissues. The infection with *A. salmonicida* did not modify this tendency in gut. Levels of TNFα1, TNFα2, IL-1β, and IL-6 became significantly higher in fish fed β-glucan supplemented diet at 6 h post infection. Such differential effects may reflect the complex interactions between the bacterium and the immunostimulant relationship with the inflammatory response of the host.	Falco, Frost, Miest, Pionnier, Irnazarow and Hoole [155]
Carp (*Cyprinus carpio*)	Not mentioned	Kidney cells incubated for 30 min.	β-Glucan stimulated the kidney derived neutrophil to produce more neutrophil extracellular traps and entrapped a significantly higher percentage of bacteria than the head kidney derived neutrophil extracellular traps.	Brogden, et al. [199]
Carp (*Cyprinus carpio*)	0–1000 μg incubated for 6, 24, and 48 h (in vitro)	pronephric primary cell culture (in vitro test)	With the concentration higher than 500 μg, MacroGard induces to a higher percentage of apoptosis in vitro.	Miest and Hoole [136]
Pacu (*Piaractus mesopotamicus*)	0.1%	15 days	The results of the present study provide additional evidence that β-glucan modulated not only the immune system, but also the release of cortisol. The β-glucan modulated cortisol levels differently after transport and after inoculation of pacu with *Aeromonas hydrophila*. Up to 24 h after transport, β-glucan increased the levels of cortisol, while in fish that were additionally inoculated with the bacterium, the elevation of the hormone levels was prevented. In inoculated fish, with reduced levels of cortisol because of β-glucan, we observed a reduction of monocytes (3 h after inoculation) and a reduction of lymphocytes as well as enhanced complement system activity (24 h).	Marinho de Mello, et al. [200]
Zebrafish (*Danio rerio*)	12.5 mg/kg BW or 0.35 g/kg of feed	14 days (after amputation)	Results showed that 1,3–1,6 β-glucans decreased fish mortality rate and enhanced both daily and cumulative regenerated fin area, independent of the ß-glucan extraction method used. Based on the mechanisms similarities of the innate immune system and tissue regeneration among different teleost species, these results may likely be extended to species of interest for the aquaculture sector.	Fronte, et al. [201]
Nile tilapia (*Oreochromis niloticus*)	100 mg/L (added in water)	8 days	Larvae that received the β-glucan treatment were ~20% heavier (10.2 mg—control; 12.3 mg—β-glucan) and ~8.5% longer (0.82 cm—control; 0.89 cm—β-glucan) compared to the control larvae.	de Jesus, et al. [202]
Carp (*Cyprinus carpio*)	0.1 μg/mL (a stock solution was prepared (0.5 g MacroGard/500 mL Milli-Q water)	14 days	The images showed significantly faster wound contraction in both treated groups compared to the control. The obtained results clearly demonstrated that β-glucan enriched bath promotes the closure of wounds in common carp and induced a local change in cytokine expression.	Przybylska-Diaz, et al. [203]
Carp (*Cyprinus carpio*)	0 1% diet or 10 mg glucan per kg body weight.	25 days	β-Glucan mediated protection against viral diseases could be due to an increased TLR-3 mediated recognition of ligands, resulting in an increased antiviral activity of macrophages.	Falco, Miest, Pionnier, Pietretti, Forlenza, Wiegertjes and Hoole [158]
Rainbow trout (*Oncorhynchus mykiss*)	0, 0.1, 0.2, and 0.5% in food	15 versus 30 days	Results suggest that spleen may be a highly responsive organ to dietary β-glucans both in healthy or infected fish, and that this organ may therefore significantly contribute to the immune reinforcement induced by such immunostimulatory diet. Our study further reveals that overdoses of β-glucans and/or prolonged medication can lead to a non-reactive physiological status and, consequently, to a poor immune response.	Douxfils, Fierro-Castro, Mandiki, Emile, Tort and Kestemont [174]
Atlantic salmon (*Salmo salar*)	1 g/kg feed	12 weeks before vaccination	Dietary supplementation decreased mortality in both unvaccinated and vaccinated *M. viscosa*-challenged fish compared to the non-supplemented groups. Similarly, mortality of infectious salmon anemia virus-challenged fish decreased from 87.5% in vaccinated fish without supplementation to 70.9% in the supplemented and vaccinated group (RPSend 26.4).	Filho, et al. [204]
Pacu (*Piaractus mesopotamicus*)	0.1% β-glucan or diet containing 1% β-glucan	7 days before inoculation	Feeding β-glucan up to 7 days significantly increased resistance against *A. hydrophila*, as well the leukocytes production and lysozyme activity of pacu suggesting benefits of the use of this immunostimulant in the farming of this species.	Biller-Takahashi, et al. [205]
Mirror carp (*Cyprinus carpio* L.)	0% (control), 0.1%, 1%, or 2% MacroGard	8 weeks	High dietary inclusion levels of β-glucan can enhance growth performance and localized intestinal leucocyte infiltration in the anterior intestine of mirror carp without detrimental effects on carcass composition, intestinal morphology, or the hemato-immunological parameters investigated.	Kuhlwein, et al. [206]
Carp (*Cyprinus carpio*)	6 mg/kg live weight	25 days	The 25-day period of β-glucan oral administration induced and enhanced an immune response in carp, and subsequent lipopolysaccharides and polyinosinic:polycytidylic acid injections significantly affected carp C-reactive protein and complement responses.	Pionnier, et al. [207]
Carp (*Cyprinus carpio*)	10 g MacroGard/kg live weight	14 days	In the present study, it is shown that feeding carp with a diet supplemented with MacroGard for a period of 2–3 weeks significantly raised the diversity and significantly altered the composition of the microbial community in the gut and, therefore, could be health promoting for this species.	Jung-Schroers, et al. [208]
Silver catfish (*Rhamdia quelen*)	0.01% of β-glucan or 0.1% of β-glucan	28 days	The addition of β-glucan to the diet improved natural complement hemolytic activity, reduced bacteremia levels and, most importantly, increased fish resistance to challenge with *A. hydrophila.*	Di Domenico, Canova, Soveral, Nied, Costa, Frandoloso and Carlos [139]
Rainbow trout (*Oncorhynchus mykiss*)	0.1 mg MacroGard/L water (bath).	14 days (as a bath)	Prolonged healing dynamics of rainbow trout muscle wounds and a very limited response to stimulation with β-glucans.	Schmidt, et al. [209]
Common carp (*Cyprinus carpio)*	25 μg/mL (in vitro study, macrophage stimulation)	-----	The identification of several candidate β-glucan receptors suggests that immune-modulatory effects of β-glucan in carp macrophages could be a result of signaling mediated by a member of the C-type lectin receptor family.	Petit, Bailey, Wheeler, de Oliveira, Forlenza and Wiegertjes [113]
Nile Tilapia (*Oreochromis niloticus)*	1 g MacroGard/kg diet	β-glucan for 4 weeks and then switching to the basal diet for 2 weeks	Tilapia continuously fed the β-glucan supplemented diets had improved weight gain and feed efficiency than those fed the control diet uninterrupted or switched from the β-glucan. Feeding tilapia β-glucan for 4 w and then switching to the basal diet for 2 w caused a significant increase in the respiratory burst, but other immune parameters were unaffected. No differences in survival to *S. iniae* infection occurred between dietary groups.	Welker, et al. [210]
Silver catfish (*Rhamdia quelen*)	0.1% (0.1 mg/L) or 0.5% (0.5 mg/L)	28 days	Results indicate that in silver catfish, wound healing occurs rapidly and improves greatly by daily bathing with β-glucan.	Dos Santos Voloski, et al. [211]
